# Emotional well-being and pain could be a greater determinant of quality of life compared to motor severity in cervical dystonia

**DOI:** 10.1007/s00702-020-02274-z

**Published:** 2020-11-04

**Authors:** Lisa Klingelhoefer, Maximilian Kaiser, Anna Sauerbier, Robert Untucht, Miriam Wienecke, Könül Mammadova, Björn Falkenburger, Olaf Gregor, K. Ray Chaudhuri, Heinz Reichmann

**Affiliations:** 1 Department of Neurology, Technische Universität Dresden, Fetscherstraße 74, Dresden, Germany; 2 Parkinson’s Foundation Centre of Excellence, King’s College Hospital, Denmark Hill, London, UK; 3 Department of Neurology, University Hospital Cologne, Cologne, Germany; 4 Department of Neurology, Klinikum Chemnitz, Chemnitz, Germany

**Keywords:** Cervical dystonia, Non-motor symptoms, Quality of life, Dystonia Non-Motor Symptoms Questionnaire, DNMSQuest

## Abstract

Non-motor symptoms (NMS) occur in patients with cervical dystonia (CD) but with variable frequencies and impact on health-related quality of life (HRQoL). To define non-motor and motor profiles and their respective impact on HRQoL in CD patients using the newly validated Dystonia Non-Motor Symptoms Questionnaire (DNMSQuest). In an observational prospective multicentre case–control study, we enrolled 61 patients with CD and 61 age- and sex-matched healthy controls (HC) comparing demographic data, motor and non-motor symptoms and HRQoL measurements. 95% CD patients reported at least one NMS. Mean total NMS score was significantly higher in CD patients (5.62 ± 3.33) than in HC (1.74 ± 1.52; *p* < 0.001). Pain, insomnia and stigma were the most prevalent NMS and HRQoL was significantly impaired in CD patients compared to HC. There was strong correlation of NMS burden with HRQoL (CDQ-24: *r* = 0.72, EQ-5D: *r* = − 0.59; *p* < 0.001) in CD patients. Regression analysis between HRQoL and NMS suggested that emotional well-being (standardized beta = − 0.352) and pain (standardized beta = − 0.291) had a major impact on HRQoL while, in contrast motor severity had no significant impact in this model. Most NMS with the exception of pain, stigma and ADL did not correlate with motor severity. NMS are highly prevalent in CD patients and occur independent of age, sex, disease duration, duration of botulinum neurotoxin therapy and socio-economic status. Specific NMS such as emotional well-being and pain have a major impact on HRQoL and are more relevant than motor severity.

## Introduction

Cervical dystonia (CD) is the commonest idiopathic focal dystonia (Albanese et al. 2013; LeDoux et al. 2016) and is one of the most frequently treated movement disorders (Defazio et al. 2013b; Group 2000). Non-motor symptoms (NMS) are common in CD and can range from pain perception, sleep dysfunction to neuropsychiatric symptoms such as depression and are considered as a research priority (Jinnah et al. 2013). However, the results from these studies vary in relation to the reported frequencies of various NMS and in their impact on health-related quality of life (HRQoL). One explanation for the reported variabilities could be related to diverse and non-standard methodologies of assessment of NMS. The Dystonia Non-Motor Symptoms Questionnaire (DNMSQuest (Klingelhoefer et al. 2019, 2020)) is a newly described 14-item self-completed questionnaire listing the presence of a range of NMS in patients with craniocervical dystonia during the past month. The overall load of the seven different relevant non-motor domains of the DNMSQuest can be calculated as an estimation of the symptoms burden [similar to NMS questionnaire for Parkinson’s disease (PD) (Chaudhuri et al. 2006)] (Klingelhoefer et al. 2019).

In this case–control study we report on the association and the impact of non-motor burden and motor symptoms on HRQoL in CD patients using the DNMSQuest.

## Methods

### Patients and healthy controls (HC)

Consecutive patients with a diagnosis of adult-onset idiopathic CD according to the definition by Albanese et al. (2013) were included in this study. To minimize the potential effect of botulinum neurotoxin (BoNT) therapy on CD and study assessments, subjects were included at the end of a BoNT therapy cycle (≥ 3 months after the last BoNT treatment). Age- and sex-matched HC were selected from unrelated companions and carers.

### Inclusion and exclusion criteria

All patients with CD and HC were included after exclusion of alternative movement disorders other than idiopathic dystonia and associated tremor. Patients with other forms of dystonia, with dementia or significant cognitive impairment [< 22 points on Montreal Cognitive Assessment (MoCA) (Dalrymple-Alford et al. 2010; Freitas et al. 2013)] and those undergoing deep brain stimulation were excluded.

### Recruitment centres

Patients were recruited from two different specialist Movement Disorders Clinics in Germany: Department of Neurology, Technical University Dresden and Department of Neurology, Klinikum Chemnitz. HC were recruited at the Department of Neurology, Technical University Dresden, Germany and at the Parkinson’s Centre and Movement Disorders Clinic, King’s College London, UK. The project fell under the auspices of the Dresden-King’s-TransCampus research initiative.

### Study assessments

The study protocol was approved by the ethics committee of the participant institutions (Dresden/Chemnitz: EK60022015, King’s College London: 15/EM/0106). All participants provided written informed consent before any study procedure was performed. The following variables were collected:

- Demographic and disease-specific variables, general medical history, medication and BoNT dosage (calculated in Dysport mouse units).
- Motor assessment: Toronto Western Spasmodic Torticollis Rating Scale (TWSTRS) (Consky et al. 1990). Unified Dystonia Rating Scale (UDRS) (Comella et al. 2003), which includes ratings for 14 body areas of motor severity and duration of dystonia with a total score of 112. As only patients with focal CD affecting the neck and shoulder/proximal arm domain have been included, the maximal total score was 24.
- Clinical Global Impression of severity (CGI-S) (Guy 1976) to assess severity of CD.
- Non-motor assessment: Dystonia Non-Motor Symptoms Questionnaire (DNMSQuest) with seven different domains: sleep, autonomic symptoms, fatigue, emotional well-being, stigma, ADL, sensory symptoms [described in Klingelhoefer et al. (2019, 2020)]; additionally a question on memory and concentration, each formulated in the same style as the DNMSQuest and referred to as cognition domain of the DNMSQuest were added; MoCA (Nasreddine et al. 2005); Beck Depression Inventory (BDI) (Beck et al. 1996).
- Quality of life questionnaires: Craniocervical dystonia questionnaire (CDQ-24) (Muller et al. 2004); EuroQol five dimensions questionnaire (EQ-5D) with index and visual analogue scale (VAS) (Group 1990).

### Statistics

As data were not normally distributed, non-parametric statistics were applied, e.g. Mann–Whitney *U* Test. Analyses were performed with Chi-Square-Test and with Spearman rank correlation coefficient (*r*). The correlation was considered: “weak” if the *r* value was < 0.3, “moderate” if 0.3–0.5, “high” if > 0.5. A *p* value of less than 0.05 indicated statistical significance. Based on the approach of van den Dool et al. (2016) a principal component analysis and subsequently a multivariable linear regression analysis in backward elimination technique was also performed. Seven different symptom complexes were defined by generating congruent topics based on motor and non-motor domains of the different scales and questionnaires (TWSTRS, UDRS, CGI-S, DNMSQuest, MOCA, BDI, CDQ-24). For each symptom complex variables were transformed to normal distributions when appropriate and standardised into Z scores to enable direct comparison. Reliability analyses were performed to control the internal consistency of the variables within each symptom complex. A Cronbach’s alpha of ≥ 0.7 was evaluated as acceptable (Cronbach 1951).

## Results

61 patients with CD and 61 HC were studied. Demographics, socio-economic status, general medical and family history are summarized in Table 1. Disease-specific characteristics of CD patients and therapy are summarized in Table 2. There was no missing data in the included study participants. Five HC and four CD patients were excluded due to neurological co-morbidities, MOCA < 22 points, segmental or multifocal dystonia.

**Table 1:** Demographics, socio-economic status and general medical history of patients with cervical dystonia and healthy controls

	Patients with cervical dystonia	Healthy controls	*p* value
Total number (*N*)	61	61	
% of female (*N*)	67.2 (41)	77 (47)	0.23 (Chi Square-Test)
Age (mean ± SD, years)	58.3 ± 13.18	61.45 ± 15.32	0.12 (MWU-Test)
Age range: minimum–maximum (years)	25–87	24–87	
Education: E1/E2/E3 (%)	31.1/42.6/26.6	19.7/32.8/47.5	**< 0.05** (Chi-Square-Test)
Years of formal education (mean ± SD, years)	10.67 ± 2.27	12.25 ± 4.03	**< 0.05** (MWU-Test)
Profession in % (*N*)			Fisher’s exact-Test
P1 if < 60 years of age	71.4 (25/35)	87.0 (20/23)	0.69
P2 if > 60 years of age	73.1 (19/26)	86.8 (33/38)	0.23
Social status: S1/S2/S3/S4 (%)	54.1/19.7/9.8/16.4	78.7/14.8/3.3/3.3	**< 0.05** (Fisher’s exact-Test)
Concomitant diseases in % (*N*)	86.9 (53)	68.9 (42)	**< 0.05** (Chi Square-Test)
Depression and anxiety disorders	18 (11)	3.3 (2)	**<** **0.001** (Chi-Square-Test)
Participants on antidepressants/anxiolytics in % (*N*)	26.2 (16)	3.3 (2)	**<** **0.001** (Chi-square-Test)
Family history in % (*N*):			Chi-square-Test
Tremor	27.9 (17)	9.8 (6)	**< 0.05**
Dystonia	6.6 (4)	0.0 (0)	**< 0.05**
Associated tremor in % (*N*):			*T* Test
Head tremor	68.9 (42)	0.0 (0)	**< 0.05**
Upper limb tremor	6.6 (4)	0.0 (0)	**< 0.05**

Bold values are statistically significant *p* < 0.05

*SD* standard deviation, Education: *E1* elementary school, *E2* high school, *E3* university. Profession: *P1* employed or autonomous, *P2* retired. Social status: *S1* married, *S2* single, *S3* widow, *S4* separated or divorced

**Table 2:** Disease specific characteristics and therapy of patients with cervical dystonia

	Patients with cervical dystonia
Disease duration (mean ± SD, years) Range: minimum–maximum (years)	12.63 ± 11.28 0–50.12
Duration of BoNT therapy (mean ± SD, years)	10.16 ± 8.83
Positive effect of BoNT therapy (mean ± SD, weeks) Range: minimum–maximum (weeks)	8.45 ± 3.88 0.00–16.00
Duration between last BoNT injection and study assessments (mean ± SD, weeks)	13.08 ± 3.56
Total dose of BoNT (mean ± SD, Dysport MU) Range: minimum–maximum (Dysport MU) BoNT type	638 ± 354 40–1800 A: 93%

Bold values are statistically significant *p* < 0.05

*SD* standard deviation, *BoNT* botulinum neurotoxin, *MU* mouse unit

### Motor symptoms

CD patients had a mean TWSTRS sum score of 34.5 ± 12.94 (range 9.5–65.5) and a mean UDRS sum score of 9.5 ± 2.81 (range 1.5–14.0). CD patients ranged from CGI score 2–7 with 77% of patients presenting with “mildly ill” (score 3) to “markedly ill” (score 5) (median ± SE 4.00 ± 0.15, minimum 2, maximum 7). In contrast, 92% of HC reported to be “normal, not at all ill” in the CGI score (median ± SE 1.00 ± 0.06, IQR 1–1, minimum 1, maximum 3). Based on the Col-Cap concept (Reichel 2013), only 6.6% of the CD patients had a malposition in one whereas 47.5% in two and 45.9% in three anatomical planes. We found no significant correlation of motor severity (assessed by TWSTRS, UDRS, CGI) with age, sex, disease duration, duration of BoNT therapy, concomitant diseases as well as operations or traumata and socio-economic status in the investigated CD patients (*p* < 0.05).

### Non-motor symptoms

**Fig. 1:**
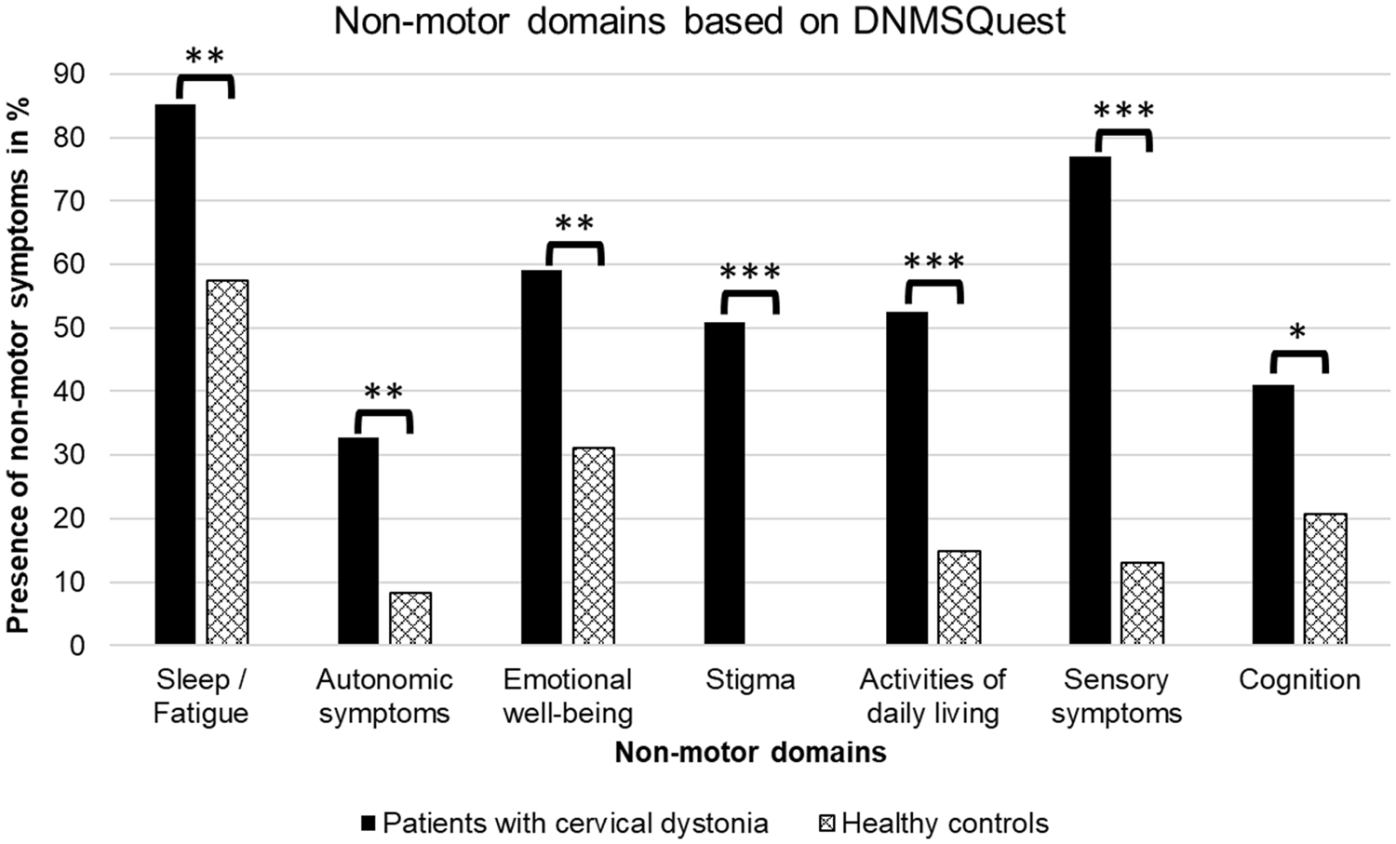
Presence of non-motor symptoms in percentage categorised in different domains assessed by Dystonia Non-Motor Symptoms Questionnaire (DNMSQuest) in patients with cervical dystonia and healthy controls (Chi-Square-Test, *p* values *p < 0.05; **p < 0.01; ***p < 0.001)

NMS were significantly more common in CD patients compared to HC with pain, insomnia and stigma being the most prevalent symptoms. Mean total NMS score was significantly higher in CD patients (5.62 ± 3.33; range 0–14) in comparison to HC (1.74 ± 1.52; range 0–6) (*p* < 0.001). 95.1% CD patients reported at least one NMS and therefore only 4.9% did not suffer from any of the assessed NMS. About 29.5% CD patients presented with at least eight NMS. Additionally, all DNMSQuest domains were significantly more prevalent in CD patients than in HC (Fig. 1). There was no impact of age, sex, disease duration, duration of BoNT therapy and socio-economic status on the assessed NMS by DNMSQuest in the investigated CD patients (*r* = − 0.14 to 0.08, *p* > 0.05). In contrast, we found a significant correlation of NMS burden in HC with age (*r* = 0.35) and concomitant diseases (*r* = 0.36) on a moderate level (*p* < 0.01).

### Association of motor and non-motor symptoms

The DNMSQuest score did not correlate with the TWSTRS I and UDRS but correlated significantly on a moderate to high level with its NMS parts (TWSTRS II: *r* = 0.43, III: *r* = 0.54, *p* < 0.001) as did the CDQ-24 (TWSTRS II: *r* = 0.54, III: *r* = 0.51, *p* < 0.001).

#### Sensory symptoms/pain

There was a linear correlation of the sensory symptoms domain of the DNMSQuest and the pain domain of the CDQ-24 and TWSTRS. The disability score (TWSTRS II) significantly correlated with pain [corresponding domains of DNMSQuest (*r* = 0.27, *p* < 0.05) and CDQ-24 (*r* = 0.62, *p* < 0.01)]. Further, pain assessed by TWSTRS III significantly correlated on a moderate level with emotional well-being (corresponding domains of DNMSQuest and CDQ-24: *r* = 0.30–0.36, *p* < 0.05) and with BDI (*r* = 0.39, *p* < 0.05). In contrast, there were only few significant correlations on a weak to moderate level of pain domains (TWSTRS III: *r* = 0.28, CDQ-24: *r* = 0.3–0.37, *p* < 0.05; DNMSQuest *p* > 0.05) with pure motor severity assessed by TWSTRS I and UDRS. In relation to motor severity, most relevant seemed to be the duration of head deviation (TWSTRS IB, UDRS I: *r* = 0.26–0.39) and the duration of BoNT effectiveness as a higher duration of head deviation and a shorter time of BoNT effectiveness were associated with more pain.

#### Emotional well-being

**Fig. 2:**
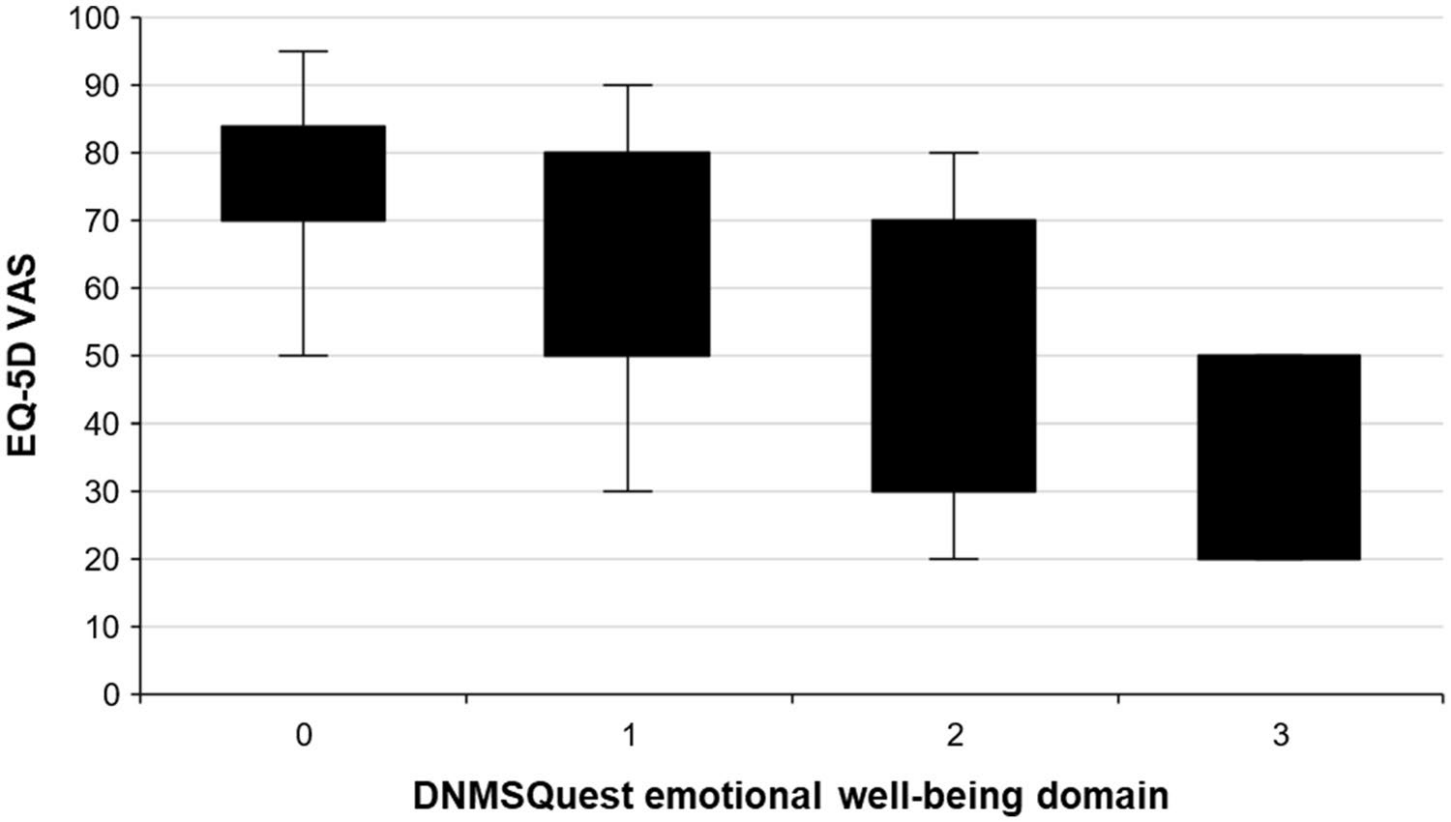
Worse emotional well-being in the Dystonia Non-Motor Symptoms Questionnaire (DNMSQuest) is associated with reduced health related quality of life (assessed by EuroQol five dimensions questionnaire visual analogue scale (EQ-5D VAS)) in patients with cervical dystonia. Boxes: 25 and 75 percentiles. Whiskers: minimum and maximum values

CD patients reported symptoms of depression, anxiety or flat moods twice as frequently as HC (Fig. 1). The mean BDI score was 8.6 ± 6.7 (range 0–24) and revealed a mild depression in 37% of CD patients. The BDI correlated on a moderate to high level with the DNMSQuest (*r* = 0.51, *p* < 0.01), the CDQ-24 (*r* = 0.67, *p* < 0.001) and the CDQ-24 emotional well-being domain (*r* = 0.4, *p* < 0.05). There was no association of the emotional well-being domain of the DNMSQuest and of the CDQ-24 with motor severity of CD (TWSTRS I, UDRS). In contrast, the emotional well-being domain of the DNMSQuest and of the CDQ-24 correlated significantly with various NMS (e.g. stigma, ADL, sleep/fatigue, pain, cognition) on a moderate to high level (*r* = 0.34–0.58, *p* < 0.05). CD patients reported a reduced HrQoL in EQ-5D VAS associated with worse emotional well-being assessed by the DNMSQuest (*p* < 0.001, Fig. 2).

#### Stigma

51% of CD patients declared the feeling of stigmatisation (Fig. 1). CD patients younger than 60 years of age suffered more often from stigmatisation than those older than 60 years (60% vs. 38.5%). The general impression of the CD assessed by CGI correlated on a high level with the stigma domain of the DNMSQuest (*r* = 0.57, *p* < 0.001) and on a moderate level with the one of the CDQ-24 (*r* = 0.3, *p* < 0.05). Stigmatisation as declared in DNMSQuest was significantly associated with worse motor severity (TWSTRS I: *r* = 0.26, *p* < 0.05), general NMS burden (*r* = 0.56, *p* < 0.001) and HrQoL (EQ-5D VAS *r* = − 0.45, CDQ-24 *r* = 0.53, *p* < 0.001).

#### Sleep/fatigue

The sleep/fatigue domain of the DNMSQuest correlated on a moderate to high level with the pain (*r* = 0.51, *p* < 0.001), the ADL (*r* = 0.5, *p* < 0.001) and the emotional well-being (*r* = 0.44, *p* < 0.001) domain of the CDQ-24. Furthermore, CD patients with anxiolytic or antidepressant medication presented with significant higher values in the DNMSQuest sleep/fatigue domain.

#### Cognition

CD patients showed a significantly lower mean MoCA value in comparison to HC (CD: 27.8 ± 1.95, range 22-30; HC: 28.5 ± 1.41, range 25–30; *p* < 0.05). In any of the single MoCA domains, CD patients were slightly worse but without significant difference to HC. Additionally, significantly more CD patients than HC declared impaired cognition and attention in the cognition domain of the DNMSQuest (41% vs. 21%). The cognition domain of the DNMSQuest significantly correlated with the emotional well-being (*r* = 0.34, *p* < 0.01) and the ADL (*r* = 0.42, *p* < 0.001) domain of the CDQ-24 as well as with the cognition domain (*r* = − 0.36, *p* < 0.01) of the MoCA.

#### Activities of daily living

With increasing limitations in ADL as assessed by the DNMSQuest, CD patients reported reduced HRQoL (CDQ-24, CDQ-24 ADL domain, *p* < 0.001), higher disability (TWSTRS II, *p* < 0.001) and had worse motor symptoms (TWSTRS I, UDRS, *p* < 0.01).

### Health-related quality of life

**Fig. 3:**
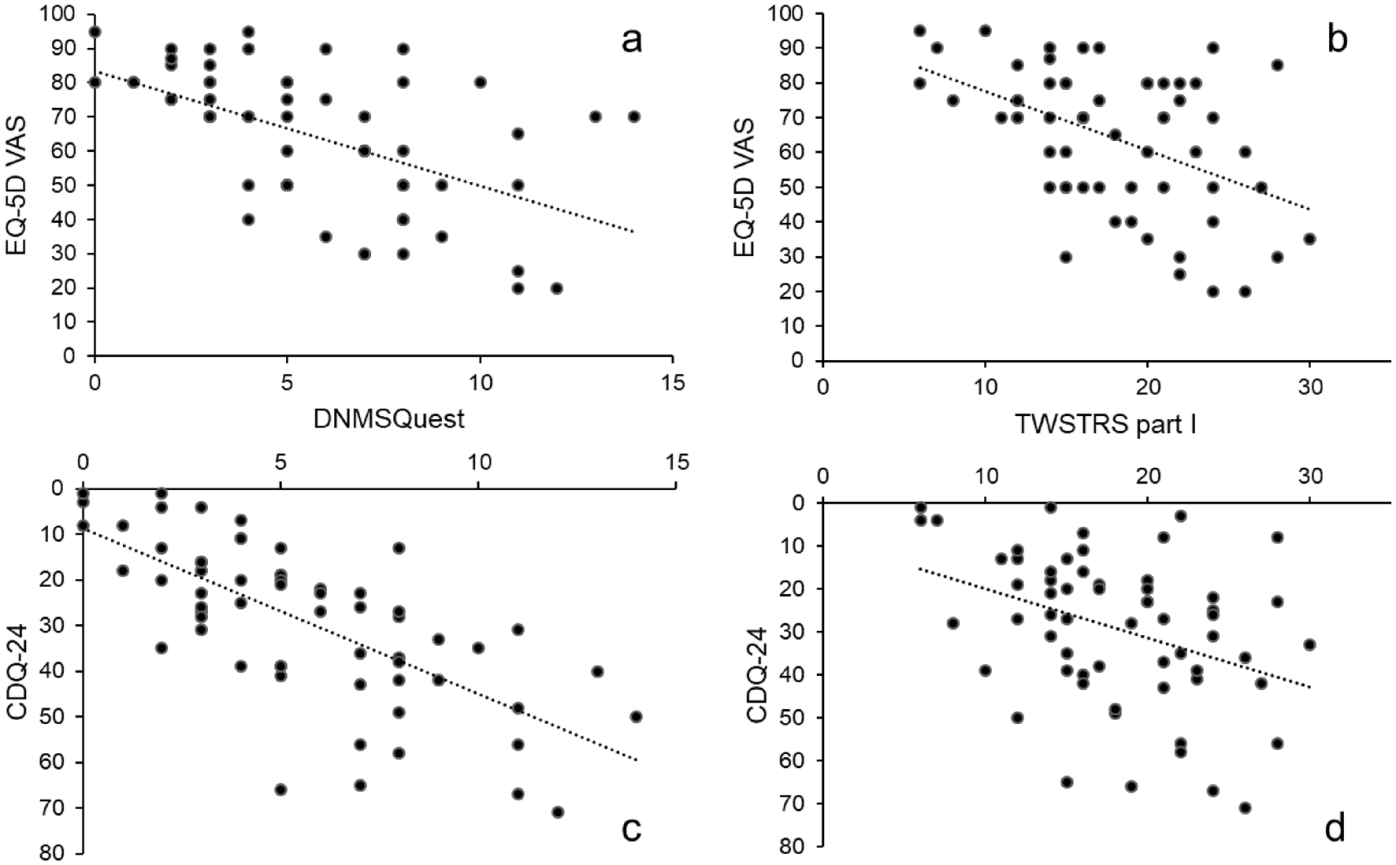
Scatterplots with negative regression lines for relationship between non-motor symptoms assessed by Dystonia Non-Motor Symptoms Questionnaire (DNMSQuest; **a**, **c**) and motor symptoms assessed by TWSTRS I (**b**, **d**) with health related quality of life measures in patients with cervical dystonia. *EQ-5D VAS* EuroQol five dimensions questionnaire visual analogue scale, *CDQ-24* craniocervical dystonia questionnaire

CD patients presented with a significantly reduced HRQoL compared to HC (EQ-5D index: CD 0.86 ± 0.18, HC 0.95 ± 0.06, *p* < 0.001; EQ-5D VAS: CD 64.2 ± 20.21, HC 73.8 ± 13.31, *p* < 0.05; CDQ-24: CD 29.11 ± 17.48). Both, the EQ-5D index and VAS correlated significantly on a high level with pure NMS assessment by DNMSQuest (index *r* = − 0.58, VAS: *r* = − 0.59, *p* < 0.001) (Fig. 3a). Further, all domains of the DNMSQuest, despite the cognition domain, correlated significantly with EQ-5D index and VAS (*r* = − 0.26 to − 0.56, *p* < 0.05). Interestingly, only the EQ-5D VAS correlated significantly on a moderate level with pure motor assessments (TWSTRS I: *r* = − 0.43, UDRS: *r* = − 0.31; *p* < 0.01) but not the EQ-5D index (Fig. 3b). Additionally, DNMSQuest total score correlated significantly on a high level with the CDQ-24 total score and its domains (*r* = 0.51–0.72, *p* < 0.001, except social/family life: *r* = 0.32, *p* < 0.05) (Fig. 3c). Also, TWSTRS I correlated significantly with CDQ-24 but on a moderate level (*r* = 0.38, *p* < 0.01, Fig. 3d).

To evaluate the impact of the defined symptom complexes on HRQoL, multiple regression analysis was used with EQ-5D VAS as dependent variable (Table 3). All symptom complexes revealed an acceptable Cronbach’s alpha. The model revealed significance [adjusted *R*^2^ 0.573, *F* (4–55) = 20.81, *p* < 0.001] and 57% of the variance of HRQoL assessed by EQ-5D VAS could be explained. Major impact on HRQoL had the symptom complexes emotional well-being followed by pain. Stigma and motor severity showed an impact but were not significant in this model.

**Table 3:** Multiple regression analysis of different symptom complexes defined by various questionnaires and scales in patients with cervical dystonia

Symptom complex	Questionnaires and scales	*B* (95% CI)	Beta	*p* value
Constant		64.490 (61.071–67.909)		**<** **0.001**
Emotional well-being	DNMSQuest emotional well-being domain, CDQ-24 emotional well-being domain, CDQ-24 social/family domain^a^	− 8.691 (− 14.391 to (− 2.991))	− 0.352	**<** **0.01**
Pain	TWSTRS part III, DNMSQuest sensory symptoms domain, CDQ-24 pain domain	− 7.057 [− 11.797 to (− 2.316)]	− 0.291	**<** **0.01**
Stigma	DNMSQuest stigma domain, CDQ-24 stigma domain	− 4.785 (− 10.099 to 0.529)	− 0.205	0.08
Motor severity	TWSTRS part I, UDRS, CGI	− 3.811 (− 7.986 to 0.364)	− 0.175	0.07
Cognition	DNMSQuest domain cognition, MoCA	Deleted by backward elimination technique		
Activities of daily living	DNMSQuest ADL domain, CDQ-24 ADL domain, TWSTRS part II	Deleted by backward elimination technique		

Bold values are statistically significant *p* < 0.05

Dependent variable: EQ-5D visual analogue scale

*EQ-5D* EuroQol five dimensions questionnaire, *B* unstandardized coefficient, *beta* standardized coefficient, *95% CI* 95% Confidence Interval, *DNMSQuest* Dystonia Non-Motor Symptoms Questionnaire, *CDQ-24* Craniocervical dystonia questionnaire, *TWSTRS* Toronto Western Spasmodic Torticollis Rating Scale, *MoCA* Montreal Cognitive Assessment, *ADL* activities of daily living

^a^The Beck Depression Inventory failed to be included in this symptom complex

## Discussion

In this multicenter observational case–control study, we describe the non-motor and motor profiles as well as their impact on HRQoL in CD patients in comparison to age- and sex-matched HC using the newly validated DNMSQuest.

The majority of investigated CD patients has intermediate motor severity, a combination of different dystonic postures and dystonic head tremor as previously described (Chan et al. 1991; Defazio et al. 2013a; Jankovic et al. 1991; Jost et al. 2020; Tomic et al. 2016; van den Dool et al. 2016; Werle et al. 2014). Importantly, our study cohort consisted of mainly middle-aged females and thus is representative for the CD population (Defazio et al. 2013b; Group 2000). Furthermore, patients of all disease durations and in all CGI stages were included.

NMS were significantly more common in CD patients with 95% reporting at least one NMS; in line with other publications (Klingelhoefer et al. 2014; Smit et al. 2017a). While in HC the NMS burden only became relevant with increasing age and comorbidities, in CD patients, the investigated NMS were independent of age, sex, disease duration, duration of BoNT therapy and socio-economic status. Further, there was no association of general NMS burden and motor severity.

Pain is common in dystonia (57–89% of CD patients), mainly in the neck and shoulder area (Comella and Bhatia 2015; Klingelhoefer et al. 2019; Sheehy and Marsden 1980; Tinazzi et al. 2020; Werle et al. 2014). In our study pain was reported by 77% of CD patients and there was a strong correlation between pain and other NMS, especially emotional well-being and depression whereas the correlation between pain and motor severity was low. This observation is therefore of interest as we also report that pain is only partly related to motor severity with the duration of head deviation being the most relevant factor. Our overall results support observations in relation to lack of correlation between pain and motor severity (Kutvonen et al. 1997; Novaretti et al. 2019). Emotional well-being, comprising symptoms of depression and anxiety, was highly prevalent and reported by 59% of CD patients in DNMSQuest; in line with other observations (Comella and Bhatia 2015; Fabbrini et al. 2010; Smit et al. 2016). Even though 37% of CD patients fulfilled the DSM criteria of mild depression (Beck et al. 1996; Kühner et al. 2007) only half of the CD patients had a documented diagnosis of depression/anxiety disorder and only a quarter was treated with anxiolytics or antidepressants. Our results indicate a marked underreporting of psychiatric comorbidities in CD patients. This is of special interest as our study and other publications reported no association of emotional well-being and motor severity (Gundel et al. 2001; Skogseid et al. 2007; Slawek et al. 2007) emphasising the need of recognition and appropriate treatment of NMS. Perceived Stigma was prevalent in around half of the investigated CD patients, especially in younger adults. Stigmatisation was mainly related to worse motor severity but also to a higher NMS burden. In our study, CD patients reporting stigmatisation were more likely to present with depressive symptoms and be treated with antidepressants or anxiolytics as described previously (Lewis et al. 2008; Papathanasiou et al. 2001). Sleep dysfunction was the second most common NMS with various presentation (66% of CD patients reporting insomnia, 41% a feeling of impaired sleep quality, 49% daytime sleepiness/fatigue). These findings are in line with other studies reporting impaired sleep in 44–72% of CD patients (Avanzino et al. 2010; Klingelhoefer et al. 2014; Paus et al. 2011). Sleep impairment was associated with reduced emotional well-being and more often reported by CD patients treated with anxiolytics or antidepressants. Importantly, sleep problems were not related to motor severity. Furthermore, our study revealed for the first time that CD patients with sleep problems had a higher pain burden than those with regular sleep and pain symptoms were reported more often during the day. Overall, sleep dysfunction and pain seem to aggravate each other independent of motor severity of CD and point out the need of specific treatment.

CD patients were significantly more impaired in their ADLs than HC. Relevant influencing factors on ADL were mainly other NMS like pain, emotional well-being, sleep dysfunction and autonomic symptoms but also motor severity. Van den Dool et al. (2016) showed that psychiatric features and pain have the largest contribution to disability in CD patients. We confirm these findings with more than half of our CD patients reporting pain as a source of disability.

NMS have a relevant influence on HRQoL in movement disorders, especially in PD (Martinez-Martin et al. 2011). In dystonia, NMS and their impact on HRQoL is topical (Ben-Shlomo et al. 2002; Camfield et al. 2002; Paracka et al. 2020; Pekmezovic et al. 2009; Smit et al. 2017b). The present study reports the impact of NMS burden, assessed by DNMSQuest, on HRQoL in CD patients (Fig. 3). HRQoL was measured by CDQ-24, a specific validated self-reported questionnaire for the assessment of HRQoL in craniocervical dystonia (Fig. 3c) and by EQ-5D, a more general validated self-reported questionnaire for the assessment of HRQoL in any kind of disease (Fig. 3a).

Based on the defined symptom complexes, emotional well-being had the highest impact on HRQoL followed by pain. Emotional well-being was also found relevant for HRQoL in CD patients in other studies (Ben-Shlomo et al. 2002; Drexel et al. 2020; Muller et al. 2002; Skogseid et al. 2007; Slawek et al. 2007). Pain as the second most relevant factor for HRQoL in the investigated CD patients, explained 41% of reduced HRQoL in a study by Werle et al. (2014). Stigma and motor severity were closely linked and relevant for HRQoL but were not significant in this study. Stigma was reported as the most relevant factor for HRQoL when using the CDQ-24 (Muller et al. 2004; van den Dool et al. 2016). This is in line with the finding of Drexel et al. reporting reduced HRQoL even under BoNT treatment (Drexel et al. 2020). Further NMS such as sleep problems and impaired ADL were associated with reduced HRQoL in this and previous studies (Smit et al. 2016, 2017b; Soeder et al. 2009; Tomic et al. 2016; Wagle Shukla et al. 2016) but were not significant within our regression model.

The potential limitations of this study are

1. The symptom complex “sleep/fatigue” covers the sleep and fatigue domain of the DNMSQuest even though fatigue is not a sleep-related problem. There are no disease-specific sleep/fatigue scales in dystonia but “sleep/fatigue” are clustered together in other NMS measures, e.g. the Non-Motor Symptom assessment scale for PD (Chaudhuri et al. 2007). Thus we used the combined symptom complex in our analysis.
2. The symptom complex “cognition” should be evaluated with caution as participants with a history of dementia or evidence of significant cognitive impairment were excluded. Nevertheless, the greater impairment of cognition and attention in CD patients compared to HC, both in subjective and objective evaluation, is an interesting finding, supported by other investigations (Czekoova et al. 2017) and needs further investigation.
3. The effect of BoNT therapy cannot be evaluated as study assessments were performed at the end of a BoNT treatment cycle (Table 2). However, we observed that a shorter positive BoNT effect is associated with a higher amount of NMS and a longer positive BoNT effect is associated with better HRQoL. This may provide a hint that BoNT has a positive effect on NMS, at least on those NMS secondary to motor symptoms. These observations need to be studied further, e.g. by re-assessment of NMS at the most effective time point after BoNT treatment. An assessment of de-novo patients would also be desirable to fully exclude any BoNT effects.

In summary, we report that NMS are highly prevalent in CD patients with 95% reporting at least one NMS. Pain, sleep problems, stigma, anxiety and depression being most common NMS. Furthermore, NMS are present throughout the whole course of dystonia and appear independent of age, sex, disease duration, duration of BoNT therapy and socio-economic status. In general, NMS burden and especially emotional well-being and pain have a major impact on HRQoL which may be a greater determinant of QoL compared to the motor symptoms in CD patients. Further, most NMS with the exception of pain, stigma and ADL occurred independent of motor severity of CD. Therefore, in line with previous publications, our data suggests that most NMS could be a primary phenomenon of dystonia. This is of major importance as a diagnostic consideration and for a holistic treatment approach of CD patients. In contrast to the diagnostic criteria for PD including NMS both as supportive and as exclusion criteria (Postuma et al. 2015), the current definition of dystonia does not consider any NMS (Albanese et al. 2013). The DNMSQuest therefore, could be an useful screening tool empowering CD patients to declare relevant NMS and thus personalise and improve holistic care.
